# Genomic and Pangenomic Insights into *Aeromonas salmonicida* subsp. *oncorhynchi* subsp. nov.

**DOI:** 10.3390/pathogens14060523

**Published:** 2025-05-23

**Authors:** Nihed Ajmi, Muhammed Duman, Hilal Ay, Izzet Burcin Saticioglu

**Affiliations:** 1 Department of Aquatic Animal Diseases, Graduate School of Health Science, Bursa Uludag University, Bursa 16059, Türkiye; nihed.ajmi.95@gmail.com; 2 Department of Aquatic Animal Diseases, Faculty of Veterinary Medicine, Bursa Uludag University, Bursa 16059, Türkiye; mduman@uludag.edu.tr; 3 Department of Molecular Biology and Genetics, Faculty of Arts and Science, Yildiz Technical University, Istanbul 34220, Türkiye; hilal.ay@yildiz.edu.tr

**Keywords:** *Aeromonas salmonicida*, antimicrobial resistance, MLPA, pangenome, subspecies nova

## Abstract

The strain A-9^T^, isolated from *Oncorhynchus mykiss* (rainbow trout) in a Turkish aquaculture facility, was characterized through integrated phenotypic, phylogenetic, and genomic analyses. Whole-genome sequencing revealed a 5.21 Mb circular chromosome (GC content: 58.16%) and three plasmids encoding proteins for mobilization and toxin–antitoxin systems. Multilocus phylogenetic analysis (MLPA) using seven housekeeping genes supported the distinct lineage of A-9^T^. Digital DNA–DNA hybridization (77.6–78.6%) and average nucleotide identity values (96.59–97.58%) confirmed taxonomic divergence from all currently recognized *A. salmonicida* subspecies. Comparative proteomic and pangenomic analyses identified 328 strain-specific genes, including virulence factors, secretion system components (Type II and Type VI), and efflux-related proteins. Although genes encoding Type III secretion systems and biofilm formation were absent, A-9^T^ harbored a broad virulence gene repertoire and resistance determinants, including *OXA-956*, *cphA5*, and *FOX-20*, supporting a multidrug-resistant phenotype. Based on its genomic, phenotypic, and functional distinctiveness, we propose the novel taxon *Aeromonas salmonicida* subsp. *oncorhynchi* subsp. nov. (type strain A-9^T^ = LMG 33538^T^ = DSM 117494^T^), expanding the taxonomic landscape of the *A. salmonicida* complex and offering insights into fish-associated bacterial evolution.

## 1. Introduction

The genus *Aeromonas* comprises a diverse group of Gram-negative, facultatively anaerobic, rod-shaped bacteria commonly found in aquatic environments [1]. Among its species, *Aeromonas salmonicida* is a significant bacterial pathogen in aquaculture, known for causing furunculosis and other infections in various fish species [2]. This species is traditionally classified into five subspecies: *A. salmonicida* subsp. *salmonicida*, *A. salmonicida* subsp. *achromogenes*, *A. salmonicida* subsp. *masoucida*, *A. salmonicida* subsp. *smithia*, and *A. salmonicida* subsp. *pectinolytica*. Each subspecies exhibits distinct genetic profiles, pathogenic properties, and environmental adaptations, leading to variations in virulence, host specificity, and antibiotic resistance [3,4,5].

Among these, *A. salmonicida* subsp. *salmonicida*, primarily associated with furunculosis in salmonids such as *Salmo salar* and *Oncorhynchus mykiss*, is the most well-characterized taxon [6]. Its pathogenicity is largely driven by the type III secretion system (T3SS), which facilitates the delivery of effector proteins into host cells, modulating immune responses and enhancing bacterial survival [7,8]. Additionally, concerns regarding antibiotic resistance have been raised due to the acquisition of multidrug-resistant plasmids, which complicate treatment efforts in aquaculture settings [9].

While the other subspecies, including *A. salmonicida* subsp. *achromogenes* and *A. salmonicida* subsp. *masoucida*, have been documented in various host species and environmental conditions, their pathogenicity mechanisms remain less understood. *A. salmonicida* subsp. *smithia* and *A. salmonicida* subsp. *pectinolytica* have demonstrated unique metabolic adaptations, with the latter being well known for its pectinolytic activity and potential saprophytic lifestyle [10]. Despite these classifications, genomic and phenotypic variations within *A. salmonicida* continue to be uncovered, indicating that additional subspecies or unique lineages may exist.

This study aims to characterize a novel *A. salmonicida* subspecies through detailed phenotypic, biochemical, and genomic analyses. By assessing its genetic distinctiveness in comparison to established subspecies, we seek to clarify its taxonomic placement and examine features of relevance to aquaculture and fish health.

## 2. Materials and Methods

### 2.1. Sampling

The bacterial strains used in this study were obtained from the culture collection of the Department of Aquatic Animal Diseases, Faculty of Veterinary Medicine, Bursa Uludağ University. Strain A-9^T^ was originally isolated in 2015 from a 40 g rainbow trout (*Oncorhynchus mykiss*) collected from a commercial fish farm in Rize, Türkiye, during routine health monitoring.

The fish was sampled as part of a routine health monitoring program at a commercial aquaculture facility. Clinical signs at the time included mild skin discoloration and reduced appetite, without any pathognomonic features. Affected fish were also observed resting near the bottom of the tank and showing little response to feed. No internal lesions were detected during necropsy. Based on these general findings, strain A-9^T^ was isolated from the internal organs, and no other bacterial species were recovered at the time of sampling. The frozen stock of strain A-9^T^ was retrieved from −80 °C in 20% glycerol and subcultured onto trypticase soy agar (TSA; Merck, Darmstadt, Germany) and blood agar (BA; Merck, Darmstadt, Germany) for further analysis. This procedure followed standardized diagnostic approaches for fish pathogens as outlined by Austin [11], emphasizing culture-based identification from fresh material using appropriate media and incubation protocols. The plates were incubated at 28 °C for 24–48 h. For long-term preservation, bacterial cultures were supplemented with 20% (*v*/*v*) glycerol and stored at −80 °C.

### 2.2. Genome Sequencing and Analysis

Genomic DNA from strain A-9^T^ was extracted using the NucleoSpin Microbial DNA Kit (Macherey–Nagel, Düren, Germany) following the manufacturer’s protocol. For long-read sequencing, 400 ng of high-molecular-weight genomic DNA was used without fragmentation, following the Oxford Nanopore Technologies (ONT) PromethION protocol. Libraries were generated using the Oxford Nanopore Ligation Sequencing Kit (SQK-NBD114-24, Oxford, UK), loaded onto a PromethION Flow Cell, and sequenced on a P2 Solo system for 24 h. In addition, the short-read sequencing libraries were prepared with the Nextera XT DNA Library Preparation Kit (Illumina, San Diego, CA, USA) and sequenced on an Illumina NovaSeq 6000 platform in 2 × 150 bp paired-end mode, utilizing a 1000-cycle HiSeq reagent kit.

Raw reads underwent quality control and preprocessing using Trim Galore (https://www.bioinformatics.babraham.ac.uk/projects/trim_galore/, accessed on 12 February 2025) (v0.6.5dev) to remove adapter sequences and low-quality bases [12]. Hybrid de novo genome assembly was performed using the Unicycler pipeline (v0.4.6) [13] on the BV-BRC (Bacterial and Viral Bioinformatics Resource Center) online platform (https://www.bv-brc.org/, accessed on 12 February 2025) [14]. The final draft genome was submitted to the GenBank, and contigs exceeding 1000 bp were annotated using the NCBI Prokaryotic Genome Annotation Pipeline (PGAP) [15].

Genomic analyses were conducted to determine taxonomic placement and genomic similarity. The digital DNA–DNA hybridization (dDDH) analysis was performed using the Type Strain Genome Server (TYGS, https://tygs.dsmz.de/, accessed on 20 February 2025), allowing comparison between strain A-9^T^ and reference type strains from the DSMZ database [16]. Additionally, the average nucleotide identity (ANI) values were calculated using both BLASTN-based (ANIb) and MUMMER-based (ANIm) algorithms via the JSpeciesWS tool (https://jspecies.ribohost.com/jspeciesws/, accessed on 20 February 2025), providing a quantitative measure of genome similarity [17].

### 2.3. Multilocus Phylogenetic Analysis (MLPA)

Seven conserved housekeeping genes, *gyr*B, *gyr*A, *rec*D, *rpo*D, *dna*J, *dna*X, and *atp*D, were extracted from the genome of strain A-9^T^. The 16S rRNA gene was intentionally excluded due to its poor discriminatory power at the intra-species level within the genus *Aeromonas*, primarily resulting from high sequence conservation and intragenomic microheterogeneities [18,19]. Sequences were aligned against reference strains using the MAFFT algorithm to ensure accurate homologous site comparison. Phylogenetic relationships were reconstructed using maximum likelihood (ML) and Neighbor-Joining (NJ) methods within MEGA X, employing the Jukes–Cantor model to estimate nucleotide substitution rates. The ML tree incorporated a gamma-distributed rate variation model (+G) and an invariable sites parameter (+I) to account for evolutionary rate differences. To ensure the reliability of inferred relationships, bootstrap analysis with 1000 replicates was conducted, providing statistical support for the resulting tree topology [20].

### 2.4. Comparative and Functional Genome Analysis

For the phylogenetic placement of strain A-9^T^, a gene-based phylogenomic analysis was conducted using the Bacterial Genome Tree Service available on the BV-BRC platform (https://www.bv-brc.org/app/PhylogeneticTree, accessed on 25 February 2025) [14]. Genome sequences of *Aeromonas* type strains and closely related species were retrieved from GenBank, representing diverse aquatic environments and host organisms. A core-genome phylogenetic tree was constructed based on 1000 single-copy orthologous protein-coding genes, following the methodology described by Olson et al. [21]. Multiple sequence alignment was performed using MAFFT v7.526 [22], and phylogenetic inference was carried out using RAxML v8.2.12 [23], employing the JTT amino acid substitution model and 100 bootstrap replicates via the fast bootstrapping algorithm. The resulting maximum likelihood tree, built from 339,023 aligned amino acid positions, provided a high-resolution comparative framework for strain A-9^T^ within the *Aeromonas* genus. Tree visualization and annotation were performed using iTOL (Interactive Tree of Life, https://itol.embl.de/, accessed on 1 March 2025) [24], incorporating metadata such as host origin, environmental source, and geographic distribution. Branch lengths were scaled to represent substitutions per site, with a tree scale of 0.01.

A comparative genomic analysis was also performed across multiple *A. salmonicida* subspecies. The genome of strain A-9^T^ was sequenced as part of this study, while reference genomes for *A. salmonicida* subsp. *achromogenes* JCM 7875^T^, *A. salmonicida* subsp. *masoucida* NBRC 13784^T^, *A. salmonicida* subsp. *pectinolytica* 34mel^T^, *A. salmonicida* subsp. *salmonicida* CIP 103209^T^, and *A. salmonicida* subsp. *smithia* CIP 104757^T^ were retrieved from the National Center for Biotechnology Information (NCBI) database. These reference genomes were included in a comparative framework with strain A-9^T^ for analyses involving virulence gene content, antibiotic resistance determinants, and proteome-level features. The functional genome characterization of strain A-9^T^ was conducted using multiple bioinformatics pipelines to predict virulence factors, antibiotic resistance genes, and other functional traits. Virulence-associated genes were identified by screening the genome against the Virulence Factor Database (VFDB) v5 (https://www.mgc.ac.cn/VFs/main.htm/, accessed on 25 February 2025) [25], with genes categorized into toxins, secretion systems, adhesion factors, and immune evasion mechanisms. Antibiotic resistance genes were detected using the Comprehensive Antibiotic Resistance Database (CARD) (https://card.mcmaster.ca/analyze, accessed on 25 February 2025) [26], through the Resistance Gene Identifier (RGI) tool, applying default parameters to classify resistance determinants by mechanism of action, including efflux pumps, antibiotic-modifying enzymes, and target alteration proteins.

For comparative pangenome analysis, the genomes of A-9^T^ and five closely related subspecies were uniformly re-annotated using Prokka v1.14.6 [27] on the Galaxy platform [28], ensuring consistent gene prediction across all datasets. The predicted protein sequences were analyzed using two complementary approaches. First, Roary v3.13.0 [29] was used to construct the pangenome, applying a 95% amino acid identity threshold for protein clustering. Genes were categorized into core (present in all genomes), soft-core (present in five genomes), shell (present in two to four genomes), and cloud (present in only one genome) components based on their distribution across the dataset. The resulting presence/absence matrix was used to identify gene clusters exclusive to individual strains. In parallel, orthologous clustering was performed using the OrthoVenn3 web server (https://orthovenn3.bioinfotoolkits.net/, accessed on 5 March 2025) [30] which employs the OrthoFinder algorithm to identify orthologous clusters through the sequence similarity of the predicted proteomes. Intersections among clusters were visualized using the UpSet plot implemented in OrthoVenn3 to facilitate the interpretation of shared and strain-specific genes. The genes identified as singletons in OrthoVenn3 were further functionally annotated using the eggNOG-mapper v2 web server (http://eggnog-mapper.embl.de, accessed on 5 March 2025) [31], which provided COG classifications, gene ontology terms, and functional domain annotations.

To further explore protein-level conservation and divergence, a comparative proteome analysis was performed using the BV-BRC Proteome Comparison Tool (https://www.bv-brc.org/, accessed on 2 March 2025) [14]. The annotated proteome of *A. salmonicida* subsp. *oncorhynchi* strain A-9^T^ was compared against the reference proteomes of the five *A. salmonicida* subspecies. The analysis was carried out using a 90% sequence identity threshold and an e-value cutoff of 1 × 10^−5^, allowing the classification of proteins into bidirectional best hits (BBHs), unidirectional best hits (UBHs), and strain-specific unique proteins. This approach enabled the identification of conserved core proteins and potential subspecies-specific protein functions unique to A-9^T^. In addition, structural variations such as gene insertions or rearrangements were assessed using Circos-based genome plots [32], highlighting syntenic regions and genomic plasticity between A-9^T^ and its closest relatives. These results provided complementary insights to the pangenome analysis, reinforcing the evolutionary distinctiveness of strain A-9^T^.

### 2.5. Morphological, Biochemical, and Physiological Tests

The morphological characteristics of strain A-9^T^ were examined by assessing colony appearance on TSA. Gram-staining was performed using a commercial kit (bioMérieux, Marcy l’Etoile, France). To analyze cellular morphology in greater detail, transmission electron microscopy (JEM 1220, JEOL, Tokyo, Japan) was used following negative staining with 2% (*w*/*v*) uranyl acetate on carbon-coated copper grids.

#### 2.5.1. Biochemical Features

To determine the enzymatic and metabolic properties of strain A-9^T^, a series of biochemical tests were performed. Oxidase activity was assessed using the filter paper method [33], a colony from a fresh 18- to 24-h culture was transferred onto filter paper pre-soaked with 1% Kovács oxidase reagent. A dark purple coloration within 5 to 10 s indicated a positive result, whereas delayed reactions (60–90 s) were recorded as weakly positive, and no color change after two minutes was considered negative. Catalase activity was evaluated using the tube method; a small portion of strain A-9 was transferred into a 12 × 75 mm test tube containing 4–5 drops of 3% (*v*/*v*) hydrogen peroxide using a wooden applicator stick to prevent agar contamination [34]. Immediate effervescence confirmed a positive reaction, while the absence of bubbles was recorded as negative. A magnifying glass was used to observe weak reactions.

Further biochemical characterization of strain A-9^T^ included tests for motility, indole production, and hydrogen sulfide (H_2_S) generation, which were analyzed using Sulfide-Indole-Motility (SIM) medium (Merck, Darmstadt, Germany). The metabolic profile was examined using Hugh and Leifson’s oxidative/fermentative (O/F) medium (HiMedia, Mumbai, India) to distinguish between oxidative and fermentative metabolic pathways. The hydrolytic activity of the strain was evaluated using different substrate-based media. DNase agar (HiMedia, Mumbai, India) was used to assess DNA degradation, while lipase activity was determined on R2A agar (BD Difco, Franklin Lakes, NJ, USA) supplemented with 1% (*w*/*v*) Tween 20 or Tween 80 (HiMedia, Mumbai, India). The hydrolysis of complex macromolecules was tested using starch agar (1% *w*/*v*; HiMedia, Mumbai, India) for polysaccharides, gelatin agar (1% *w*/*v*; Merck, Darmstadt, Germany) for gelatin liquefaction, skimmed milk agar (3% *w*/*v*; HiMedia, Mumbai, India) for casein hydrolysis, L-tyrosine agar (0.5% *w*/*v*; Merck, Darmstadt, Germany) for amino acid metabolism, and bile aesculin agar (BAA; Merck, Darmstadt, Germany) to evaluate esculin hydrolysis.

To characterize further the metabolic capabilities of strain A-9^T^, biochemical profiling was conducted using API 20NE and API 20E test strips (bioMérieux, Marcy l’Etoile, France) and BIOLOG GENIII MicroPlates (Biolog, Hayward, CA, USA). These assays were incubated at 28 °C for 24–48 h, following the manufacturers’ instructions.

#### 2.5.2. Growth Characteristics and Environmental Tolerance

The growth characteristics of strain A-9^T^ were examined under various conditions. Growth was assessed on multiple media, including nutrient agar (NA; Merck, Darmstadt, Germany), R2A agar (BD Difco, Franklin Lakes, NJ, USA), bile aesculin agar (BAA; Merck, Darmstadt, Germany), MacConkey agar (MC; Merck, Darmstadt, Germany), seawater agar (SwA; HiMedia, Mumbai, India), brain heart infusion agar (BHIA; Merck, Darmstadt, Germany), TSA , thiosulfate–citrate–bile salts–sucrose agar (TCBS; Oxoid, Basingstoke, UK), marine agar (MA; BD Difco, Franklin Lakes, NJ, USA), and 5% (*v*/*v*) sheep blood agar (BA; Merck, Darmstadt, Germany).

The ability of strain A-9^T^ to grow under anaerobic conditions was evaluated using the AnaeroPack system (Mitsubishi Gas Chemical Co., Inc., Tokyo, Japan), with incubation for up to two weeks. The temperature tolerance of strain A-9^T^ was assessed by culturing on TSA at 0–50 °C in 5 °C increments to determine its growth range and optimal temperature.

### 2.6. Assessment of Biofilm Formation and Antibiotic Susceptibility

Biofilm formation was evaluated using the crystal violet microtiter plate assay. Strain A-9^T^ was initially grown in Trypticase Soy Broth (TSB; Merck, Darmstadt, Germany) for 24 h, followed by a 1:100 dilution of the overnight cultures. The bacterial suspension was adjusted to 0.5 McFarland in fresh TSB, and 100 µL of the diluted culture was transferred into a 96-well plate. Wells containing only TSB served as negative controls. The plate was incubated at 28 °C for 24 h. After incubation, wells were washed with phosphate-buffered saline (PBS) to remove non-adherent cells and then stained with 200 µL of 1% crystal violet for one hour at room temperature. Excess stain was removed by washing with PBS, followed by the addition of 200 µL of 95% ethyl alcohol and incubation for 15 min. The optical density (OD) was measured at 600 nm using a Thermo Multiskan GO microplate reader (Thermo Fisher Scientific, Waltham, MA, USA), and biofilm formation was categorized as follows: no formation (OD test < OD control), weak formation (OD control < OD test < 2 × OD control), moderate formation (2 × OD control < OD test < 4 × OD control), or strong formation (OD test > 4 × OD control). All experiments were conducted in triplicate to ensure accuracy and reproducibility [35,36].

The antimicrobial susceptibility of A-9^T^ was assessed using the disc diffusion method in accordance with CLSI VET03 guidelines for aquatic bacteria [37]. The antibiotic panel included the following: Enrofloxacin (5 µg), Flumequine (30 µg), Ciprofloxacin (5 µg), Doxycycline (30 µg), Oxytetracycline (30 µg), Florfenicol (30 µg), Trimethoprim/Sulfamethoxazole (1.25/23.75 µg), Amoxicillin (25 µg), Amoxicillin/Clavulanic Acid (30 µg), Ampicillin (10 µg), Erythromycin (15 µg), Oxolinic Acid (2 µg), and 0/129 (2,4-Diamino-6,7-di-iso-propylpteridine phosphate). Mueller–Hinton Agar (MHA) plates were inoculated with **100 µL** of bacterial suspensions adjusted to 0.5 McFarland standard. Antibiotic discs were applied to the agar surface, and plates were incubated at 28 °C for 24–28 h. Zones of inhibition were measured in millimeters (mm), and strains showing no measurable zone (Ø) were interpreted as resistant. *Aeromonas salmonicida* subsp. *salmonicida* ATCC 33658 served as the quality control strain. Interpretations were validated according to CLSI VET03/VET 04-S2 breakpoints, where applicable [37].

## 3. Results

### 3.1. Phenotypic Characteristics

Strain A-9^T^ exhibited classic features of the genus *Aeromonas*. The cells were Gram-negative, aerobic, non-motile rods, with dimensions ranging from 0.9 to 1 µm in width and 2 to 2.1 µm in length. On tryptic soy agar (TSA), colonies were cream-colored, convex, circular, and had smooth surfaces with entire margins. Transmission electron microscopy revealed that strain A-9^T^ is rod-shaped and possesses a single polar flagellum (Figure S1). The isolate tested positive for both oxidase and catalase activities.

A summary of phenotypic features differentiating strain A-9^T^ from its closest phylogenetic relatives is provided in Table 1. Comprehensive phenotypic data for the strain are detailed in Table S1.

### 3.2. Biofilm Formation and Antibiotic Susceptibility

Biofilm formation by strain A-9^T^ was evaluated via crystal violet staining and quantified spectrophotometrically at 600 nm. The optical density (OD_600_) value for A-9^T^ was 0.4624, while the negative control (only TSB) yielded an OD_600_ of 0.5735. The resulting OD ratio (0.806) fell below the OD of the negative control, indicating no biofilm formation under the tested conditions. This suggests that the strain lacks the ability to adhere to surfaces and develop structured biofilm communities.

Antibiotic susceptibility testing was performed using the disc diffusion method, and results were interpreted according to the EUCAST guidelines, where applicable. As standardized breakpoints for *Aeromonas* spp. are only available for cefepime, ceftazidime, aztreonam, ciprofloxacin, levofloxacin, and trimethoprim-sulfamethoxazole, the susceptibility of strain A-9^T^ to these antibiotics was compared to the EUCAST zone diameter breakpoints. Based on these criteria, A-9^T^ was susceptible to trimethoprim-sulfamethoxazole, but showed intermediate susceptibility to ciprofloxacin. For other tested antibiotics that lack the established EUCAST breakpoints, strain A-9^T^ exhibited measurable inhibition zones for florfenicol (37 mm), doxycycline (30 mm), oxytetracycline (33 mm), enrofloxacin (26 mm), flumequine (14 mm), and erythromycin (17 mm), suggesting varying levels of antimicrobial activity. In contrast, A-9^T^ was resistant to amoxicillin, amoxicillin/clavulanic acid, ampicillin, oxolinic acid, and 0/129, as no inhibition zones were detected. While definitive susceptibility classifications could not be assigned for all antibiotics due to the absence of formal breakpoints, the results indicate that strain A-9^T^ exhibits intrinsic resistance to β-lactams and oxolinic acid, while maintaining susceptibility to several fluoroquinolones and tetracyclines (Table S1).

### 3.3. Genome Analysis

The complete genome of strain A-9^T^ has been deposited in GenBank under accession number CP178319.1. The genome comprises a 5,197,168 bp circular chromosome and three linear plasmids, 7284 bp (CP178320.1), 5635 bp (CP178321.1), and 4616 bp (CP178322.1), yielding a total assembly size of 5,214,703 bp. The assembly is highly contiguous, with an N50 of 5,197,168 bp and an L50 of 1. Annotation was performed using the NCBI Prokaryotic Genome Annotation Pipeline (PGAP). A total of 4888 genes were identified, including 4724 coding sequences (CDSs), all of which encode proteins. The genome also includes 164 RNA genes, comprising 11 copies of 5S rRNA, 10 copies of 16S rRNA, and 10 copies of 23S rRNA, along with 128 tRNA genes and 5 non-coding RNAs (ncRNAs). A total of 96 pseudogenes were annotated, including 35 frameshifted, 56 incomplete, 16 with internal stop codons, and 8 with multiple annotation issues.

**Figure 1:**
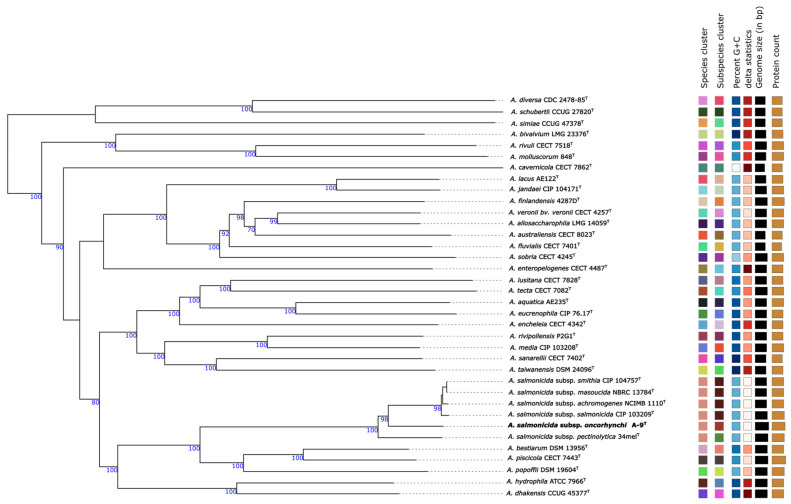
Phylogenetic tree of *Aeromonas* strains based on Genome BLAST Distance Phylogeny (GBDP) analysis using TYGS. The tree was inferred with FastME 2.1.6.1 using GBDP formula d5, with branch lengths scaled accordingly. Bootstrap values > 60% (100 replicates) are shown at nodes. The tree is midpoint-rooted for clarity. Colored squares denote species and subspecies clusters; GC content, genome size, and predicted protein counts are displayed. GC variations > 1% may indicate taxonomic inconsistencies. Delta statistics (δ values) summarize overall tree structure.

**Figure 2:**
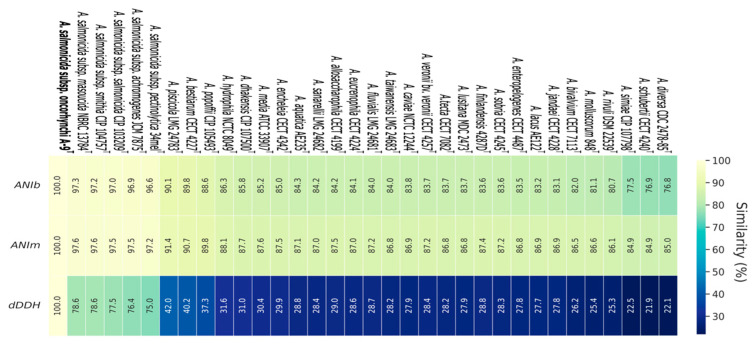
Heatmap representation of the genetic similarity among various *Aeromonas* species, including newly identified strains (A-9^T^). The heatmap illustrates pairwise similarity percentages, with a color gradient transitioning from dark shades (lower similarity) to lighter shades (higher similarity), providing a visual representation of genetic relationships. Numerical values indicate the calculated similarity percentages for quantitative interpretation.

Genome-based phylogenetic analysis was conducted using the Type Strain Genome Server (TYGS), and the resulting phylogenomic tree showed that strain A-9^T^ clusters closely with *A. salmonicida* subspecies, with an average branch support value of 98.8% (Figure 1). The digital DNA-DNA hybridization (dDDH) analysis revealed that strain A-9^T^ shares the highest dDDH values with *A. salmonicida* subsp. *masoucida* NBRC 13784^T^ (78.6%), *A. salmonicida* subsp. *smithia* CIP 104757^T^ (78.6%), and *A. salmonicida* subsp. *salmonicida* CIP 103209^T^ (77.5%), while showing lower dDDH values (<42%) with other *Aeromonas* species (Figure 2). Since the dDDH threshold for species delineation is 70%, these results strongly suggest that strain A-9^T^ belongs to the *A. salmonicida* species complex.

Comparative ANI (average nucleotide identity) analysis further supported these findings. The ANIb values ranged from 96.59% to 97.27%, while the ANIm values ranged from 97.22% to 97.58%, with the highest similarities observed to *A. salmonicida* subsp. *masoucida* (97.27% ANIb; 97.58% ANIm) and *A. salmonicida* subsp. *smithia* (97.21% ANIb; 97.57% ANIm). Since ANI values above 95% indicate the same species, these results further confirm that A-9^T^ belongs to *A. salmonicida* (Figure 2).

Together, these findings suggest that strain A-9^T^ is a member of *A. salmonicida*, most closely related to *A. salmonicida* subsp. *masoucida* and *A. salmonicida* subsp. *smithia*. However, its exact subspecies designation requires further phenotypic and functional characterization to determine if it represents an existing subspecies or a novel taxonomic unit within *A. salmonicida*.

### 3.4. MLPA-Based Phylogenetic Results

**Figure 3:**
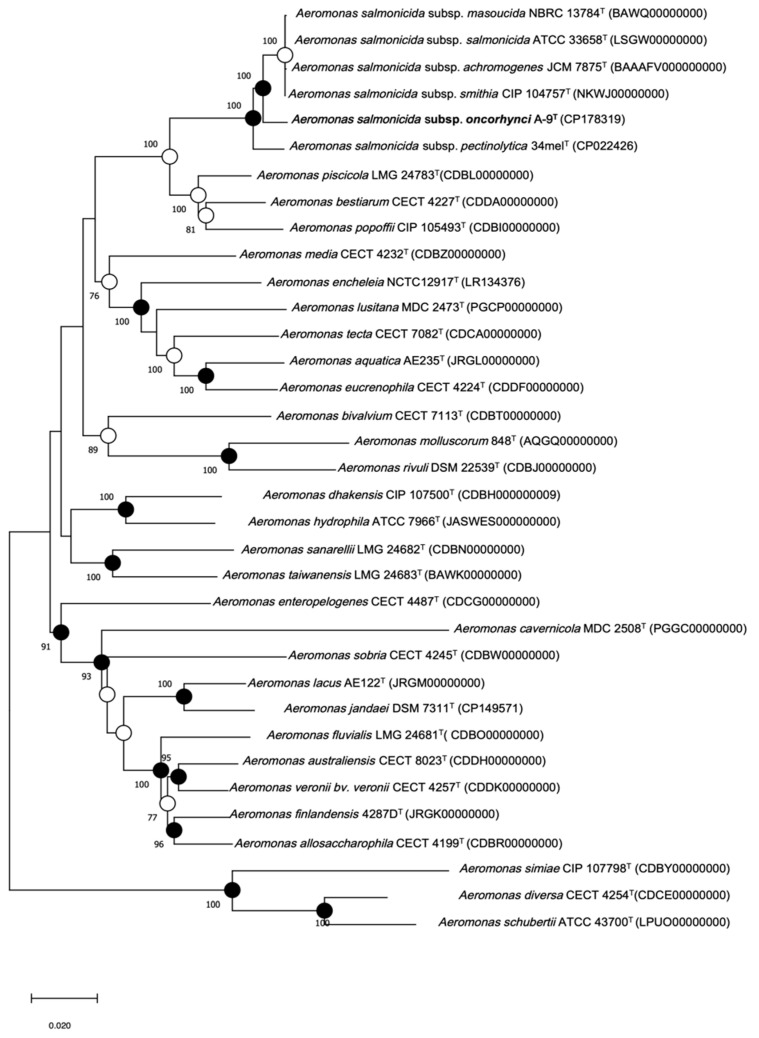
Multilocus phylogenetic tree of *Aeromonas* species based on concatenated sequences of seven housekeeping genes (*gyr*B, *gyr*A, *rec*D, *rpo*D, *dna*J, *dna*X, and *atp*D). The tree includes type and reference strains, with strain A-9^T^ highlighted in bold. Filled circles indicate nodes supported by ML, NJ, and MP methods; open circles represent support from ML or MP. Bootstrap values (>70%, 1000 replicates) are shown at nodes. Scale bar indicates 0.02 nucleotide substitutions per site.

To achieve higher phylogenetic resolution, the MLPA was performed using concatenated sequences of seven housekeeping genes (*gyr*B, *gyr*A, *rec*D, *rpo*D, *dna*J, *dna*X, and *atp*D). Phylogenetic trees were reconstructed using both the maximum likelihood (ML) and Neighbor-Joining (NJ) methods, with bootstrap values > 70% considered as strong support (Figure 3). The analysis placed strain A-9^T^ within the *A. salmonicida* species complex, clustering closely with *A. salmonicida* subsp. *masoucida* NBRC 13784^T^ and *A. salmonicida* subsp. *smithia* CIP 104757^T^. Despite this close relationship, strain A-9^T^ formed a distinct phylogenetic lineage, suggesting potential subspecies-level differentiation. The separation from other *A. salmonicida* subspecies supports its classification as a novel taxonomic unit within this clade.

### 3.5. Virulence Factors

**Figure 4:**
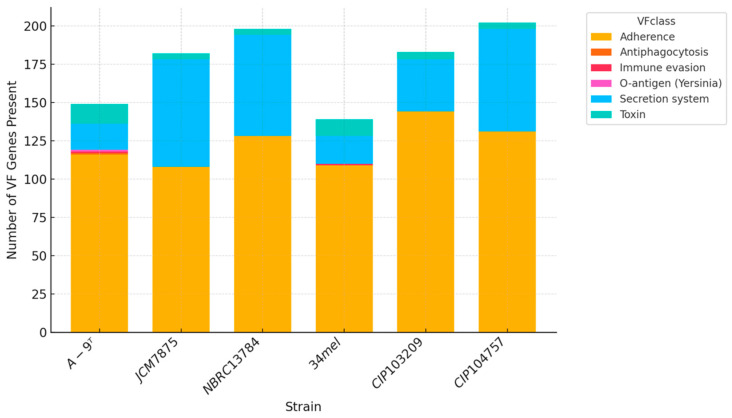
Stacked bar chart representing the distribution of virulence factor (VF) gene classes across six *A. salmonicida* subspecies genomes. The VF genes were grouped into major functional categories, namely, *Adherence*, *Secretion system*, *Toxin*, *Antiphagocytosis*, *Immune evasion*, *and O-antigen (Yersinia)*. Each bar represents a strain, with color segments indicating the number of genes identified in each VF class. Subspecies included *A. salmonicida* subsp. *oncorhynchi* strain A-9^T^, *A. salmonicida* subsp. *achromogenes* JCM 7875^T^, *A. salmonicida* subsp. *masoucida* NBRC 13784^T^, *A. salmonicida* subsp. *pectinolytica* 34mel^T^, *A. salmonicida* subsp. *salmonicida* CIP 103209^T^, and *A. salmonicida* subsp. *smithia* CIP 104757^T^.

The analysis of virulence-associated genes using the Virulence Factor Database (VFDB) revealed a diverse and expanded virulence gene repertoire in strain A-9^T^ (Table S2). The genome of strain A-9^T^ harbored a total of 149 virulence-related genes, representing the highest count among all compared *A. salmonicida* subspecies. These genes were associated with key pathogenic mechanisms, including adhesion, motility, secretion systems, and toxin production. In terms of adherence, strain A-9^T^ possessed complete operons for Flp type IV pili (*flp*1–*flp*L), Tap pili (*tap*B–*tap*W), Mannose-sensitive hemagglutinin (*Msh*) pili (*msh*A–*msh*Q), and Type I fimbriae (*fim*A–*fim*E), contributing to host colonization, biofilm formation, and environmental persistence. A full complement of genes for flagellar assembly and chemotaxis was also detected, including *fli*, *flg*, *che*, *flh*, and *mot* family members. Strain A-9^T^ carried genes encoding components of both the type II secretion system (T2SS) (*exe*A–*ex*eN) and the type VI secretion system (T6SS), including effectors and structural proteins such as *hcp*1, *vgr*G1, *vgr*G3, *vas*H, and *dot*U. Notably, type III secretion system (T3SS) genes were absent in strain A-9^T^, distinguishing it from subspecies *salmonicida* and *masoucida*, which encode T3SS components (*asc*, *aop*, *aex*, and *ati* genes). A broad toxin gene profile was also observed in A-9^T^. This included RTX toxins (*rtx*A–*rtx*H), aerolysin (*aer*A), exotoxin A (*tox*A), heat-stable enterotoxin (*ast*), hemolysin (*hly*A), and cytolytic hemolysin (ahh1), indicating a capacity for multiple cytotoxic effects, including host membrane disruption and red blood cell lysis. These genes were present either partially or entirely absent in other subspecies. For instance, *tox*A and *ast* were not identified in *A. salmonicida* subsp. *salmonicida*, *A. salmonicida* subsp. *smithia*, or *A. salmonicida* subsp. *masoucida*. Capsule-associated gene analysis showed the presence of the capsular polysaccharide biosynthesis gene *rml*C in A-9^T^. However, genes related to *Acinetobacter*-like capsule synthesis, which were detected in other subspecies, were absent. Among the analyzed subspecies, *A. salmonicida* subsp. *masoucida* NBRC 13784^T^ harbored the highest number of virulence-associated genes (198), followed by *A. salmonicida* subsp. *achromogenes* JCM 7875^T^ (172), *A. salmonicida* subsp. *smithia* CIP 104757^T^ (161), *A. salmonicida* subsp. *salmonicida* CIP 103209^T^ (154), and *A. salmonicida* subsp. *oncorhynchi* A-9^T^ (149). The lowest number was observed in *A. salmonicida* subsp. *pectinolytica* 34mel^T^, with only 139 virulence genes detected. While A-9^T^ did not carry the most extensive virulence gene set, it exhibited a unique combination of secretion systems and toxin-related genes that clearly distinguish it from the classical *A. salmonicida* lineage, supporting its characterization as a genetically and functionally distinct subspecies (Figure 4).

### 3.6. Antimicrobial Resistance Gene Analysis

The Comprehensive Antibiotic Resistance Database (CARD) analysis identified β-lactamase genes across all *A. salmonicida* subspecies evaluated. Strain A-9^T^ carried *OXA*-956, *cph*A5, and *FOX*-20, with 100%, 96.4%, and 84.82% identity to reference sequences, respectively. Strain 34mel^T^ (*A. salmonicida* subsp. *pectinolytica*) exhibited the same resistance profile, with all three genes detected at high identity values. Strains JCM 7875^T^ (*A. salmonicida* subsp. *achromogenes*) and NBRC 13784^T^ (*A. salmonicida* subsp. *masoucida*) carried *OXA*-956 and *FOX*-18, while cphA5 was also present in JCM 7875. *FOX*-18 was further identified in CIP 103209 (*A. salmonicida* subsp. *salmonicida*) and CIP 104757^T^ (*A. salmonicida* subsp. *smithia*), which also carried either *cph*A5 or *OXA*-956. Overall, β-lactamase genes from three major families (*OXA*, *Cph*A, and *FOX*) were variably distributed among the subspecies (Table 2).

### 3.7. Genome-Based Phylogeny and Subspecies Delineation

**Figure 5:**
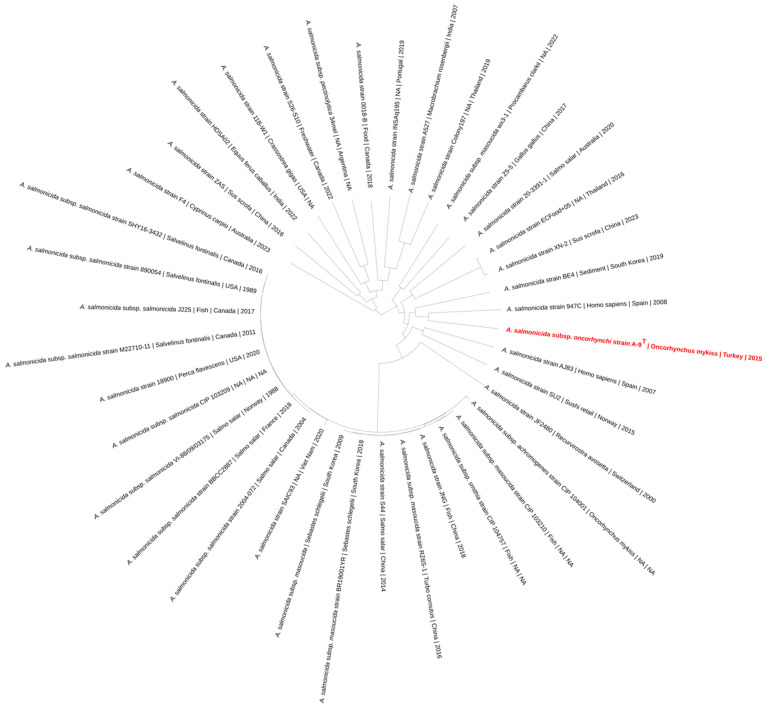
Circular phylogenetic tree of 39 *A. salmonicida* genomes based on the single-copy core genes. Tip labels include strain designation, host species, country of isolation, and collection year. Strain A-9^T^ is marked in red. The tree was annotated with metadata and visualized using Interactive Tree of Life (iTOL).

A phylogenomic tree was constructed based on 39 *A. salmonicida* genomes. The resulting topology displayed clustering patterns consistent with the established subspecies designations, including *A. salmonicida* subsp. *salmonicida*, *A. salmonicida* subsp. *achromogenes*, *A. salmonicida* subsp. *masoucida*, *A. salmonicida* subsp. *pectinolytica*, and *A. salmonicida* subsp. *smithia*. Strain A-9^T^ formed an independent branch, separate from the main clusters associated with classical subspecies. The closest genome to that of strain A-9^T^, based on branch length, was the genome of strain 947C, isolated from *Homo sapiens* (Figure 5). Strains derived from fish hosts such as *Oncorhynchus mykiss*, *Salmo salar*, and *Salvelinus fontinalis* were primarily grouped within the *A. salmonicida* subsp. *salmonicida* clade. Isolates of *A. salmonicida* subsp. *pectinolytica* and *A. salmonicida* subsp. *masoucida* formed distinct clades, while *A. salmonicida* subsp. *achromogenes* and *A. salmonicida* subsp. *smithia* appeared more dispersed. The metadata including host species, country of isolation, and year were integrated into the tree to provide contextual information.

### 3.8. Comparative Proteome Analysis

**Figure 6:**
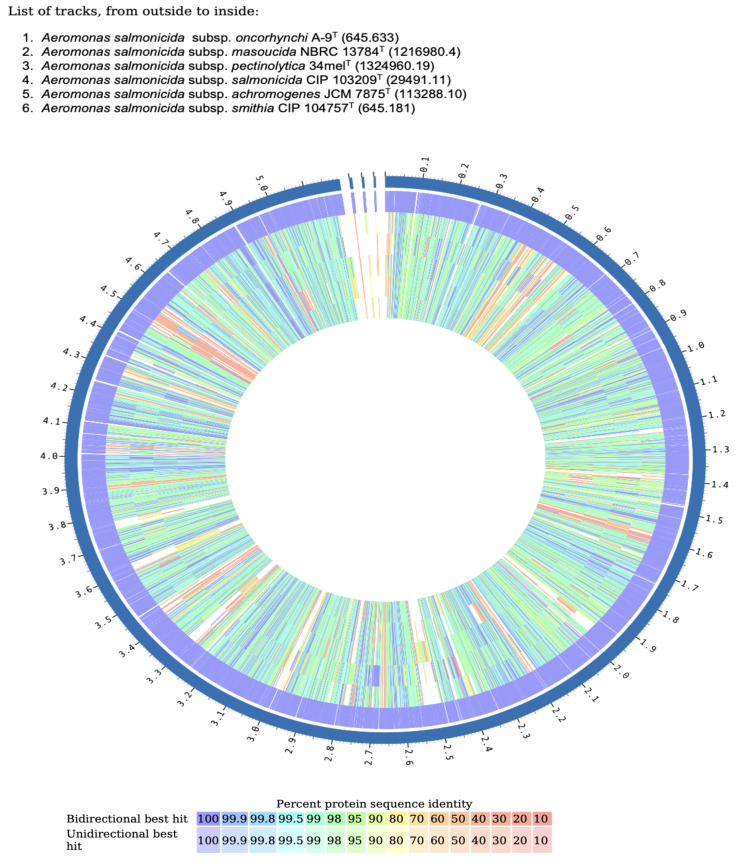
Circos plot depicting proteome comparison between *A. salmonicida* subsp. *oncorhynchi* strain A-9^T^ (outer ring) and five *A. salmonicida* subspecies. Color scale represents protein identity (from 100% to 10%) based on bidirectional and unidirectional best BLASTP hits. Tracks are ordered from outermost to innermost as follows: A-9^T^, NBRC 13784^T^, 34mel^T^, CIP 103209^T^, JCM 7875^T^, and CIP 104757^T^.

The predicted proteome of strain A-9^T^, comprising 4765 proteins, was compared with five reference *A. salmonicida* subspecies to evaluate patterns of protein conservation and divergence. A total of 328 proteins were unique to strain A-9^T^ and absent from all five compared subspecies. These included proteins associated with mobility (*pilT_1* and *pilT_2*), stress response (*yaa*A), and transcriptional regulation (*pcpR_1*), as well as a substantial number of hypothetical proteins and mobile element-related sequences. The highest number of shared proteins was observed with *A. salmonicida* subsp. *masoucida* NBRC 13784^T^ (4091 proteins), followed by *A. salmonicida* subsp. *achromogenes* JCM 7875^T^ (4061), *A. salmonicida* subsp. *smithia* CIP 104757^T^ (4056), and *A. salmonicida* subsp. *salmonicida* CIP 103209^T^ (4053). The lowest similarity was noted with *A. salmonicida* subsp. *pectinolytica* 34mel^T^, which shared 3912 proteins with strain A-9^T^. Across conserved protein clusters, pairwise sequence identity values ranged from 89% to 100%, with most orthologous proteins exhibiting high similarity in the 95–100% range. These conserved proteins, forming the core proteome, were distributed broadly across the genomes, indicating strong functional preservation. However, localized regions of reduced identity and absence were also observed, corresponding to strain-specific proteins or genomic insertions. These divergence patterns were further illustrated through a Circos-based proteome comparison (Figure 6), which visualized strain A-9^T^ as the outer reference ring. The plot displayed continuous high-identity bands across all subspecies, interspersed with gaps and low-similarity segments, particularly pronounced in *A. salmonicida* subsp. *pectinolytica* 34mel^T^ and *A. salmonicida* subsp. *salmonicida* CIP 103209^T^. These regions highlight areas of genomic plasticity, likely associated with mobile genetic elements, prophages, and environmental adaptation genes. Overall, the comparative analysis revealed a high degree of proteomic conservation within the *A. salmonicida* complex, with strain A-9^T^ maintaining a stable functional core while exhibiting distinct accessory regions. The strongest similarity to *A. salmonicida* subsp. *masoucida* supports phylogenomic findings, while observed divergence in other subspecies underscores the ecological and evolutionary uniqueness of A-9^T^.

### 3.9. Pangenome Analysis

The comparative pangenome analysis of six *A. salmonicida* isolates—strain A-9^T^ and the subspecies *Aeromonas* subsp. *achromogenes* JCM 7875^T^, *Aeromonas* subsp. *masoucida* NBRC 13784^T^, *Aeromonas* subsp. *pectinolytica* 34mel^T^, *Aeromonas* subsp. *salmonicida* CIP 103209^T^, and *Aeromonas* subsp. *smithia* CIP 104757^T^—was performed using Roary, which identified a total of 7292 orthologous gene clusters across the dataset. Among these, 3038 clusters were identified as core genes, being present in all six strains. The soft-core genome, comprising genes shared by five strains, included 422 clusters. A further 1166 clusters were categorized within the shell genome, representing genes found in two to four strains. Finally, 2666 gene clusters were identified as part of the cloud genome, defined as genes unique to a single strain.

**Figure 7:**
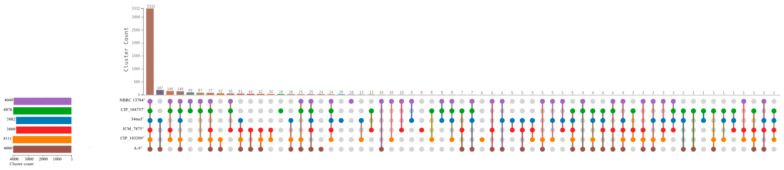
Pangenome comparison of *A. salmonicida* strains. The bar plot presents the total number of gene clusters for six *A. salmonicida* strains, each represented by a distinct color: purple for *A. salmonicida* subsp. *masoucida* (NBRC 13784^T^), green for *A. salmonicida* subsp. *smithia* (CIP 104757^T^), blue for *A. salmonicida* subsp. *pectinolytica* (34mel^T^), red for *A. salmonicida* subsp. *salmonicida* (CIP 103209^T^), orange for *A. salmonicida* subsp. *achromogenes* (JCM 7875^T^), and brown for *A. salmonicida* subsp. *oncorhynchi* (strain A-9^T^).

A breakdown of strain-specific genes revealed that *A. salmonicida* strain A-9^T^ contained the largest number of unique gene clusters (*n* = 902), followed by *A. salmonicida* subsp. *pectinolytica* 34mel^T^ (*n* = 871), *Aeromonas* subsp. *achromogenes* JCM 7875^T^ (*n* = 401), *Aeromonas* subsp. *salmonicida* CIP 103209^T^ (*n* = 374), *Aeromonas* subsp. *smithia* CIP 104757^T^ (*n* = 67), and *Aeromonas* subsp. *masoucida* NBRC 13784^T^ (*n* = 51). To confirm and visualize the distribution of shared and unique orthologous clusters, the gene presence/absence matrix was further analyzed using OrthoVenn3. The UpSet plot visualization (Figure 7) highlighted the intersections among gene clusters across strains, showing that 328 clusters were found exclusively in the genome of strain A-9^T^ and not shared with any of the other five subspecies.

The functional annotation of these 328 singleton clusters was performed using eggNOG-mapper. Annotated genes among the singleton set included membrane-associated efflux and transporter systems (*cus*A_4, *cus*A_5, *cus*B, *tol*C_3, *vap*B_1, and *vap*B_2), heavy metal response regulators (*cop*R_2 and *cus*S), stress-related proteins (*kat*E, *dps*1, and *yaa*A), and type VI secretion system-related elements (*hcp*A_1 and *hcp*A_2). Several DNA repair and recombination-related genes (*umu*C _1–4, *umu*D_1, r*ec*D2_1–7, and *xer*C_3) were also present. Polysaccharide biosynthesis and export machinery were represented by genes such as *kps*M, *kps*T, and *kps*D_1/2. Additionally, regulatory proteins (*sly*A_3, *bdc*A_2, and *tra*R_1) and sugar metabolism genes (*man*X, *man*Z, *aga*A, and *aga*C) were observed. A substantial portion of genes within this set remained functionally uncharacterized and were annotated as hypothetical proteins.

The plasmid analysis of strain A-9^T^ revealed the presence of three distinct plasmids, collectively encoding 24 proteins. These include multiple proteins involved in the replication, partitioning, mobilization, and stabilization of plasmids, such as *Rep*B replication proteins, *Par*A and *Par*G partitioning ATPases, and mobilization-related proteins from the *Mob*C, *Mbe*B, and *Mbe*D/*Mob*D families. Additionally, several toxin–antitoxin system components were identified, including proteins from the *Rel*E/*Par*E, *Abi*Ei, and *Ccd*AB families, as well as nucleotidyltransferases. The plasmids also encode proteins such as ribbon–helix–helix transcriptional regulators, serine/threonine phosphatases, and multiple hypothetical proteins with currently uncharacterized functions. All identified genes were annotated using the NCBI’s protein database, confirming their plasmid origin and relevance to plasmid maintenance and transfer. A complete list of these plasmid-encoded proteins is provided in Table S3.

### 3.10. Description of Aeromonas salmonicida *subsp.* oncorhynchi *subsp. nov.*

*Aeromonas salmonicida* subsp. *oncorhynchi* (on.co.rhyn’chi. N.L. gen. n. *oncorhynchi*, of *Oncorhynchus*, named after the rainbow trout *Oncorhynchus mykiss*, from which the type strain was isolated).

Cells are Gram-stain-negative, non-motile, aerobic, and rod-shaped, measuring 0.9–1 µm wide and 2–2.1 µm long. Colonies on tryptic soy agar (TSA) are convex, smooth, and cream-colored. The strain is oxidase-positive and catalase-positive, while H_2_S production and indole production are negative. It can grow under anaerobic conditions. Strain A-9^T^ grows on nutrient agar, R2A agar, MacConkey agar, seawater agar, brain heart infusion agar (BHIA), TSA, marine agar, and thiosulfate–citrate–bile salts–sucrose (TCBS) agar. Growth is also observed on 5% sheep blood agar, where it exhibits β-hemolysis. However, no growth is observed on bile aesculin agar. The strain grows within a temperature range of 4–45 °C and tolerates 0–4% NaCl but is not tolerant to higher salt concentrations (8% NaCl). Hydrolysis tests show positive hydrolysis for Tween 20, Tween 80, starch, gelatin, and casein, while DNase and L-tyrosine hydrolysis are negative. In the API 20NE test, the strain is positive for nitrate reduction, D-glucose fermentation, arginine dihydrolase, aesculin hydrolysis, gelatin hydrolysis, and β-galactosidase activity, but negative for indole production and urease activity. It assimilates D-glucose, L-arabinose, D-mannose, D-mannitol, N-acetyl-D-glucosamine, D-maltose, potassium gluconate, capric acid, and malic acid, but does not assimilate adipic acid, trisodium citrate, or phenylacetic acid. In the API 20E test, the strain is positive for β-galactosidase, arginine dihydrolase, lysine decarboxylase, Voges–Proskauer, gelatin hydrolysis, glucose and mannitol fermentation, sorbitol fermentation, and arabinose fermentation, but negative for ornithine decarboxylase, citrate utilization, H_2_S production, urease, tryptophan deaminase, indole production, rhamnose fermentation, sucrose fermentation, melibiose fermentation, and amygdalin fermentation. Biolog GENIII system analysis shows that the strain utilizes α-D-glucose, D-mannose, D-mannitol, dextrin, α-D-lactose, trehalose, β-methyl-D-glucoside, D-galactose, D-cellobiose, D-maltose, D-fructose, L-alanine, L-galactonic acid lactone, trehalose, D-gluconic acid, and D-glucuronic acid, but does not utilize D-raffinose, gelatin, Tween 40, sorbitol, myo-inositol, L-arginine, or L-aspartic acid. Chemical sensitivity assays indicate that the strain is resistant to vancomycin and tetrazolium blue, but sensitive to nalidixic acid and aztreonam. It tolerates 1% NaCl but does not tolerate 8% NaCl.

The type strain is A-9^T^ (=LMG 33538^T^ = DSM 117494^T^), isolated in 2015 from rainbow trout (*Oncorhynchus mykiss*) collected from a commercial fish farm in Rize, Türkiye. The complete genome sequence of strain A-9^T^ is available under GenBank accession number CP178319 and associated plasmids under accession numbers CP178320–CP178322. The 16S rRNA gene sequence is deposited under accession number PV211279.1.

## 4. Discussion

The genomic, phylogenetic, and functional analyses conducted in this study collectively support the classification of *A. salmonicida* strain A-9^T^ as a novel subspecies, here proposed as *A. salmonicida* subsp. *oncorhynchi*. This taxonomic reassignment is strongly substantiated by whole-genome-based metrics, including digital DNA–DNA hybridization (dDDH) (77.6–78.6%) and average nucleotide identity (ANI) values (96.59–97.58%), which exceed species-level thresholds while falling below established subspecies demarcation criteria (≥79–80% dDDH), as proposed by Meier-Kolthoff et al. [45]. Moreover, the multilocus phylogenetic analysis (MLPA) of seven housekeeping genes (*gyr*B, *gyr*A, *rec*D, *rpo*D, *dna*J, *dna*X, and *atp*D) confirmed the distinct evolutionary lineage of A-9^T^, separating it from the five currently recognized subspecies (*salmonicida*, *achromogenes*, *masoucida*, *smithia*, and *pectinolytica*). The MLPA approach was chosen over 16S rRNA sequencing, which, despite its widespread use, lacks sufficient resolution for distinguishing closely related *Aeromonas* taxa [46,47]. Notably, the multilocus phylogenetic analysis revealed that strain A-9^T^ clusters closely with *A. salmonicida* isolates obtained from human clinical cases in Spain. This phylogenetic proximity suggests potential host flexibility and underlines the genomic similarity between fish-associated and human-pathogenic strains of *A. salmonicida*. While strain A-9^T^ was isolated from a rainbow trout during a routine health inspection, its close evolutionary relationship to human-derived isolates may indicate shared virulence determinants or mobile genetic elements that facilitate cross-species adaptation. This observation aligns with previous reports emphasizing the zoonotic potential of *Aeromonas* species, particularly in immunocompromised individuals or through environmental exposure [20,48].

Despite the absence of hallmark type III secretion system (T3SS) genes, a key virulence determinant in *A. salmonicida* subsp. *salmonicida* and *masoucida* [49,50], strain A-9^T^ retains a diverse array of virulence factors. The VFDB-based analysis identified components of the Type II (*exe*A–*exe*N) and Type VI (*hcp*1, *vgr*G1, and *vgr*G3) secretion systems, alongside a suite of cytotoxins including RTX toxins (*rtx*A–*rtx*H), hemolysins (*hly*A and *ahh*1), aerolysin (*aer*A), and heat-stable enterotoxin (*ast*) , suggesting a compensatory mechanism of pathogenicity in the absence of T3SS. Additionally, genes encoding structural adherence factors—*Flp*-type IV pili (*flp*1–*flp*L), Msh pili (*msh*A–*msh*Q), and Type I fimbriae (*fim*A–*fim*E)—were found to support colonization, host attachment, and potential biofilm development [7,51].

Strain A-9^T^ also demonstrated a multidrug-resistant (MDR) phenotype. CARD analysis revealed the presence of beta-lactamase genes *OXA*-956 (100% identity), cphA5 (95.2%), and FOX-20 (86.09%), implicating resistance to penicillins, carbapenems, and cephalosporins. Furthermore, the detection of *Arn*T, associated with lipid A modification, points to potential resistance to polymyxins such as colistin, despite their limited use in aquaculture. These genomic findings were corroborated by phenotypic resistance to several veterinary antibiotics, notably amoxicillin, erythromycin, and trimethoprim/sulfamethoxazole, reinforcing the concern for resistance dissemination in aquaculture settings [9,52]. Despite the presence of multiple resistance and virulence-associated genes, strain A-9^T^ did not exhibit detectable biofilm formation under the tested conditions. This observation aligns with reports of biofilm variability across *Aeromonas* species, where environmental conditions, quorum sensing, and strain-specific regulatory mechanisms influence adherence capabilities [53,54]. The absence of biofilm may reflect an adaptive response to aquaculture-associated selective pressures, where alternative persistence strategies—such as stress tolerance, secretion systems, and resistance mechanisms—compensate for the lack of biofilm-based protection. Additionally, it is possible that strain A-9^T^ retains conditional biofilm potential that is only expressed under specific environmental cues, as seen in other *Aeromonas* strains [55,56]. This highlights the functional diversity and ecological plasticity of the *A. salmonicida* complex and adds further depth to the characterization of strain A-9^T^.

Distinct from the virulence plasmid pAsa5, which encodes T3SS and is often thermolabile [57,58], strain A-9^T^ carries three plasmids harboring 24 annotated proteins. These include replication proteins (*Rep*B), partitioning ATPases (*Par*A and *Par*G), mobilization genes (*Mob*C, *Mbe*B, and *Mbe*D), and toxin–antitoxin systems (*Rel*E/*Par*E, *Abi*Ei, and *Ccd*AB). This unique plasmidome likely contributes to plasmid stability, horizontal gene transfer, and niche adaptation under stress. The identification of multiple hypothetical proteins suggests additional, uncharacterized plasmid-linked functions specific to strain A-9^T^.

To further investigate genomic plasticity and functional divergence, comparative proteomic and pangenomic analyses were conducted. Proteome comparisons revealed that strain A-9^T^ shared 4091 proteins with *A. salmonicida* subsp. *masoucida* NBRC 13784^T^ but also encoded 328 unique proteins not found in any of the five compared subspecies. These unique proteins included mobility-related ATPases (*pilT_1* and *pilT_2*), oxidative stress response regulators (*yaa*A), and transcriptional factors (*pcpR_1*), suggesting functional adaptations that may support environmental sensing, survival under stress, and niche specialization. The presence of these accessory proteins reflects strain-specific flexibility beyond the conserved core proteome and may be linked to selective pressures in aquaculture environments, such as antimicrobial exposure or host immune interactions. These findings are consistent with previous studies. Elbehiry et al. [58] demonstrated that proteomic signatures can effectively differentiate closely related *Aeromonas* strains, while Li et al. [59] highlighted the role of stress-responsive proteins and regulatory systems in bacterial adaptation to hostile environments. Together, the unique proteomic profile of strain A-9^T^ supports its taxonomic distinction as a novel subspecies and provides insight into the evolutionary forces shaping subspecies diversity within the *A. salmonicida* complex.

The pangenome analysis provided further evidence of the genomic distinctiveness of strain A-9^T^ within the *A. salmonicida* complex. While all six subspecies shared a conserved set of core genes reflecting their common evolutionary origin, A-9^T^ exhibited a notably larger and more functionally diverse accessory genome. This accessory content included multiple orthogroups uniquely present in A-9^T^, as revealed by OrthoVenn3 clustering, many of which were absent from the other subspecies despite overall genomic similarity. These unique clusters encoded genes related to plasmid replication and stability, mobile genetic elements, membrane transporters, toxin–antitoxin systems, and several hypothetical proteins with potential roles in host interaction or niche adaptation. Of particular interest was the presence of gene sets involved in heavy metal and oxidative stress resistance, such as *cus*A, *cop*R, *kat*E, and *dps1*, alongside type VI secretion system components (*hcpA_1* and *hcpA_2*) and DNA repair elements (*rec*D2, *umu*C, and *xer*C). These genes are often associated with survival in fluctuating environments and microbial competition, suggesting that strain A-9^T^ may be particularly well adapted to aquaculture-associated conditions where selective pressures such as antibiotic exposure, immune challenge, and nutrient limitation are common. The detection of carbohydrate metabolism genes (*aga*A, *man*X, and *man*Z), as well as capsular biosynthesis and export machinery (*kps*M, *kps*D, and *kps*T), may further enhance this strain’s ability to colonize diverse host tissues or environmental niches. Importantly, the diversity of the accessory genome in strain A-9^T^ illustrates how genomic plasticity can drive subspecies-level divergence within a bacterial complex. As shown in recent studies by Maistrenko et al. [60] and Truong et al. [61], fluctuations in gene content, particularly through horizontal gene transfer and recombination, play a critical role in shaping bacterial ecology, pathogenic potential, and evolutionary trajectories. The distinct genetic repertoire of A-9^T^, particularly the acquisition of adaptive genes absent in closely related subspecies, highlights a genome that is both functionally innovative and ecologically specialized. This divergence not only reinforces the proteome-based findings but also supports the recognition of strain A-9^T^ as a novel subspecies with a unique evolutionary path within the *A. salmonicida* lineage.

Our study sheds light on the genomic, phenotypic, and functional features of *A. salmonicida* subsp. *oncorhynchi* subsp. nov. strain A-9^T^, isolated from rainbow trout in a Turkish aquaculture setting. Multilocus phylogenetic analysis revealed that strain A-9^T^ forms a distinct lineage within the *A. salmonicida* complex, supporting its classification as a novel subspecies. The strain harbors a range of virulence-associated genes, including components of the Type II and Type VI secretion systems, and exhibits moderate biofilm formation, alongside a well-defined plasmid profile contributing to its genomic distinctiveness. Additionally, antibiotic susceptibility testing revealed resistance to β-lactams and erythromycin, aligning with known resistance patterns in aquatic *Aeromonas* species. These findings enhance our understanding of the taxonomic and functional diversity within *A. salmonicida*, and contribute valuable genomic data for the broader study of this genus in aquatic environments.

## 5. Conclusions

This study reports *A. salmonicida* subsp. *oncorhynchi* subsp. nov., represented by strain A-9^T^ (LMG 33538^T^ = DSM 117494^T^), isolated from *Oncorhynchus mykiss* in Türkiye. A combination of genomic, phenotypic, and phylogenetic analyses established its distinction from previously described subspecies. These findings contribute to the taxonomy of the *A. salmonicida* complex and underscore the value of integrative approaches for identifying novel bacterial taxa.

## Figures and Tables

**Table 1:** Selected physiological and biochemical characteristics of A-9^T^. Note that the results in this table only show differential observations between the strains. +, positive; −, negative; ND, not defined; 1: *A. salmonicida* subsp. *oncorhynchi* A-9^T^; 2: *A. salmonicida* subsp. *smithia* CIP 104757^T^; 3: *A. salmonicida* subsp. *pectinolytica* 34mel^T^; 4: *A. salmonicida* subsp. *achromogenes* JCM 7875^T^; 5: *A. salmonicida* subsp. *salmonicida* CIP 103209^T^; 6: *A. salmonicida* subsp. *masoucida* NBRC 13784^T^.

	1	2 *	3 **	4 ***	5 ****	6 *****
Temperature (°C)	4–45 °C	4–25 °C	5–37 °C	5–30 °C	5–35 °C	5–37 °C
NaCl (%) (*w*/*v*)	0–4	0–2	ND	ND	ND	0–4
Motility	−	−	−	+	+	+
Hydrolysis of						
DNase	−	+	+	+	+	+
Tween 80	+	−	ND	+	+	ND
L-Tyrosin	−	ND	ND	ND	ND	+
Growth ability on						
McConkey Agar	+	−		+	+	ND
Indole	−	−	+	+	−	+
Anaerobic environment	+	+	ND	−	ND	+
API 20 NE						
Indole Production	−	+	+	+	−	+
Fermentation (D-Glucose)	+	+	+	−	+	+
Arginine Dihydrolase	−	−	−	+	+	+
Urease	−	−	−	+	+	+
Hydrolysis of Aesculin	+	−	−	−	+	ND
Assimilation of						ND
L-Arabinose	+	+	+	−	+	+
D-Mannose	+	−	+	+	+	+
D-Mannitol	+	−	+	+	+	+
N-acetyl-D-Glucosamine	+	−	+	ND	ND	+
D-Maltose	+	−	ND	+	+	+
Potassium Gluconate	+	ND	ND	−	−	−
API 20 E						
ONPG: Ortho-Nitrophenyl-β-galactoside (tests for β-galactosidase activity)	+	ND	+	−	−	+
IND: Indole Production	−	ND	+	+	ND	+
VP: Voges–Proskauer Test	+	ND	+	−	ND	−
SOR: Sorbitol Fermentation	+	ND	+	−	−	−
SAC: Sucrose Fermentation	+	ND	+	+	−	+
ARA: Arabinose Fermentation	+	ND	+	−		−
BIOLOG GENIII						
Carbon source utilization assays						
D-Raffinose	+	ND	−	−	−	−
D-Melibiose	+	ND	−	−	−	−
Sucrose	+	ND	+	+	−	+
D-Turanose	+	ND	−	−	−	−

Data from * Austin et al. (1989) [38]; ** Pavan et al. (2000) [39]; *** Hänninen and Hirvelä-Koski (1997) and Dalsgaard et al. (1998) [40,41]; **** Popoff (1984) and Hahnel and Gloud (1982) [42,43]; ***** Kimura (1969) [44].

**Table 2:** Antimicrobial resistance (AMR) genes identified in *A. salmonicida* subspecies based on comparative genomic analysis.

Strain	AMR Genes Detected
*A. salmonicida* subsp. *achromogenes* JCM 7875^T^	*OXA*-956, *cph*A5, *FOX*-18
*A. salmonicida* subsp. *masoucida* NBRC 13784^T^	*OXA*-956, *FOX*-18
*A. salmonicida* subsp. *pectinolytica* 34mel^T^	*OXA*-956, *cph*A5, *FOX*-20
*A. salmonicida* subsp. *salmonicida* CIP 103209^T^	*cph*A5, *FOX*-18
*A. salmonicida* subsp. *smithia* CIP 104757^T^	*OXA*-956, *FOX*-18
*A. salmonicida* subsp. *oncorhynchi* A-9^T^	*OXA*-956, *cph*A5, *FOX*-20

## Data Availability

The data presented in this study are available in the article.

## Supplementary Materials

The following supporting information can be downloaded at: https://www.mdpi.com/article/10.3390/pathogens14060523/s1, Figure S1: Transmission electron micrographs of strain A-9^T^; Table S1. Physiological and biochemical characteristics of *Aeromonas salmonicida* subsp. *oncorhynchi* strain A-9^T^; Table S2: Virulence genes in strain A-9^T^; Table S3: Plasmid-associated genes identified in strain A-9^T^.
