# Supplementary material for: Genomic and Pangenomic Insights into Aeromonas salmonicida subsp. oncorhynchi subsp. nov
Source: Pathogens. 2025 May 23;14(6):523. doi: 10.3390/pathogens14060523 (PMC12196135; doi:10.3390/pathogens14060523)
Supplement: Supplementary file 1 [file pathogens-14-00523-s001.zip › pathogens-3588659-supplementary.pdf]

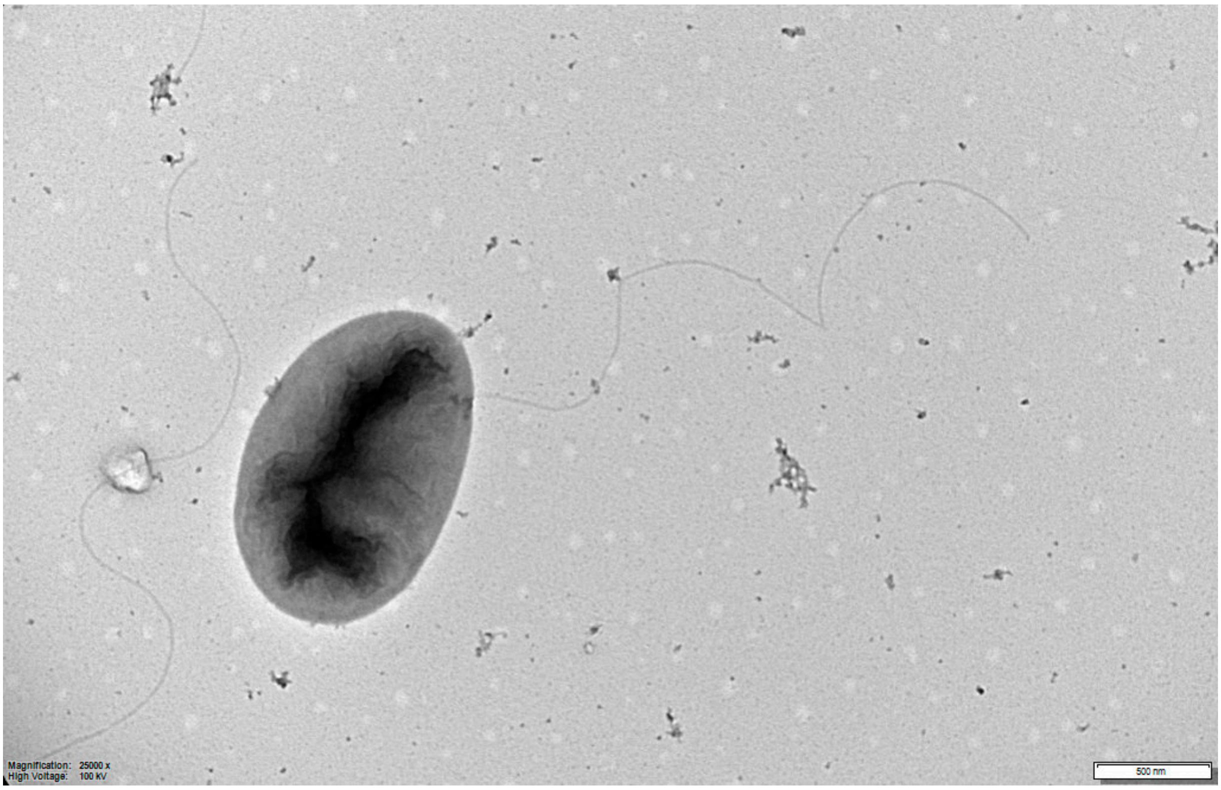

Figure S1: Transmission electron micrographs of strains A-9^{T}

Table S1. Physiological and biochemical characteristics of *Aeromonas salmonicida* subsp. *oncorhynchi* strain A-9^T

**Characteristics**	**A-9^T**
Oxidase	+
Catalase	+
Temperature (◦C)	4-45C
NaCl (%) (w/v)	0-4
Motility	-
**Hydrolysis of**	
DNase	-
Tween 20	+
Tween 80	+
Starch	+
Gelatin	+
Casein	+
L-tyrosin	-
**Growth ability on**	
Nutrient Agar	+
R2A	+
Bile Aesuculin Agar	-
McConkey Agar	+
Sea water Agar	+
Brain Heart Agar	+
TSA	+
Thiosulfate-citrate-bile salts-sucrose agar (TCBS)	+
Marine agar	+
%5 Sheep Blood Agar	β
Anaerobic environment	-
**API 20 NE**	
Reduction of Nitrate to Nitrite	+
Indole Production	-
Fermentation (D-Glucose)	+
Arginine Dihydrolase	-
Urease	-
Hydrolysis of Aesculin	+
Hydrolysis of Gelatin	+
β-galactosidase (Para-NitroPhenyl-ßDGalactopyranosidase)	+
**Assimilation of:**	
D-Glucose	+
L-Arabinose	+
D-Mannose	+
D-Mannitol	+
N-acetyl-D-Glucosamine	+
D-Maltose	+
Potassium Gluconate	+

Capric Acid	+
Adipic Acid	-
Malic Acid	+
Trisodium Citrate	-
Phenylacetic Acid	-
**API 20 E**	
Ortho-Nitrophenyl-β-galactoside (tests for β-galactosidase activity)	+
Arginine Dihydrolase	+
Lysine Decarboxylase	+
Ornithine Decarboxylase	-
Citrate Utilization	-
Hydrogen Sulfide Production	-
Urease	-
Tryptophan Deaminase	-
Indole Production	-
Voges-Proskauer Test	+
Gelatinase	+
Glucose Fermentation	+
Mannitol Fermentation	+
Inositol Fermentation	-
Sorbitol Fermentation	+
Rhamnose Fermentation	-
Sucrose Fermentation	+
Melibiose Fermentation	-
Amygdalin Fermentation	+
Arabinose Fermentation	+
**BIOLOG GENIII**	
**Carbon source utilization assays**	
D-Raffinose	+
α-D-Glucose	+
D-Sorbitol	-
Gelatin	-
Pectin	+
p-Hydroxy-Phenylacetic Acid	-
Tween 40	-
Dextrin	+
α-D-Lactose	-
D-Mannose	+
D-Mannitol	-
Glycyl-L-Proline	-
D-Galacturonic Acid	+
Methyl Pyruvate	-
γ-Amino-Butyric Acid	-
D-Maltose	+
D-Melibiose	+
D-Fructose	+

D-Arabitol	-
L-Alanine	-
L-Galactonic Acid Lactone	+
D-Lactic Acid Methyl Ester	-
α-Hydroxy Butyric Acid	-
D-Trehalose	+
β-Methyl-D-Glucoside	-
D-Galactose	+
myo-Inositol	-
L-Arginine	-
D-Gluconic Acid	+
L-Lactic Acid	-
ß-Hydroxy-D,L-Butyric Acid	-
D-Cellobiose	+
D-Salicin	-
3-Methyl Glucose	+
Glycerol	-
L-Aspartic Acid	-
D-Glucuronic Acid	+
Citric Acid	-
α-Keto-Butyric Acid	-
Gentiobiose	+
N-Acetyl-D-Glucosamine	+
D-Fucose	+
D-Glucose-6-PO4	+
L-Glutamic Acid	-
Glucuronamide	+
α-Keto-Glutaric Acid	+
Acetoacetic Acid	+
Sucrose	+
N-Acetyl-β-D-Mannosamine	+
L-Fucose	+
D-Fructose-6-PO4	-
L-Histidine	-
Mucic Acid	-
D-Malic Acid	-
Propionic acid	-
D-Turanose	+
N-Acetyl-D-Galactosamine	-
L-Rhamnose	+
D-Aspartic Acid	-
L-Pyroglutamic Acid	-
Quinic acid	-
L-Malic Acid	+
Acetic Acid	+
Stachyose	+
N-Acetyl-Neuraminic Acid	-

Inosine	-
D-Serine	-
L-Serine	+
D-Saccharic Acid	+
Bromo-Succinic Acid	-
Formic Acid	-
**Chemical sensitivity assays**	
1% NaCl	+
1% Sodium Lactate	-
Troleandomycin	-
Lincomycin	-
Vancomycin	+
Nalidixic Acid	+
Aztreonam	-
pH 6	+
4% NaCl	-
Fusidic Acid	-
Rifamycin SV	+
Guanidine HCl	+
Tetrazolium Violet	+
Lithium Chloride	-
Sodium Butyrate	-
pH 5	-
8% NaCl	-
D-serine	+
Minocycline	-
Niaproof 4	+
Tetrazolium Blue	+
Potassium Tellurite	-
Sodium Bromate	-
**Antibiotic susceptibility assays (mm)***	
Enrofloxacin (5 µg)	26
Flumequine (30 µg)	14
Ciprofloxacin (5 µg)	26
Doxycycline (30 µg)	30
Oxytetracycline (30 µg)	33
Florfenicol (30 µg)	37
Trimethoprim/sulfamethoxazole (1.25/23.75 µg)	32
Amoxicillin (25 µg)	Ø (No zone)
Amoxicillin/Clavulanic Acid (30 µg)	Ø (No zone)
Ampicillin (10 µg)	Ø (No zone)
Erythromycin (15 µg)	17
Oxolinic Acid (2 µg)	Ø (No zone)
0/129 (2,4-Diamino-6,7-di-iso-propylpteridine phosphate)	Ø (No zone)

+; Positive, **–;** negative, **W;** Weak Positive, ***;** The inhibition zones of the *Aeromonas salmonicida* subsp. *salmonicida* ATCC 33658 strain, used as a quality control strain in the disk diffusion test, were found to be within the reference range specified by the CLSI.

Table S2: Virulence Genes in strain A-9^T

Virulence Factor Class	Virulence Factor	Virulence Genes Present
**Adherence**	Flp type IV pili	flp1; flpA; flpB; flpC; flpD; flpE; flpF; flpG; flpH; flpI; flpJ; flpK; flpL
	Mannose-sensitive hemagglutinin (Msh) pilus	mshA; mshB; mshC; mshD; mshE; mshF; mshG; mshI1; mshI; mshJ; mshK; mshL; mshM; mshN; mshO; mshP; mshQ
	Polar flagella;	cheA-2; cheB-2; cheR-3; cheV; cheW; cheY; cheZ; flaA; flaB; flaG; flaH; flaJ; flgA; flgB; flgC; flgD; flgE; flgF; flgG; flgH; flgI; flgJ; flgK; flgL; flgM; flgN; flhA; flhB; flhF; flhG; fliA; fliE; fliF; fliG; fliH; fliI; fliJ; fliK; fliL; fliM; fliN; fliO; fliP; fliQ; fliR; flmD; flmH; flrA; flrB; flrC; maf-1; maf-2; motX; motY; nueA; nueB; pomA2; pomA; pomB2; pomB
	Tap type IV pili;	tapB; tapC; tapD; tapF; tapM; tapN; tapO; tapP; tapQ; tapT; tapU; tapV; tapW; tapY1; tppA; tppB; tppC; tppD; tppE; tppF
	Type I fimbriae;	fimA; fimC; fimD; fimE
**Secretion system;**	T2SS;	exeA; exeB; exeC; exeD; exeE; exeF; exeG; exeH; exeI; exeJ; exeK; exeL; exeM; exeN
	T6SS;	hcp1; vgrG1; vgrG3
**Toxin;**	Aerolysin AerA/Cytotoxic enterotoxin Act	aerA/act
	Extracellular hemolysin AHH1	ahh1
	Heat-stable cytotonic enterotoxin	ast
	Hemolysin HlyA	hlyA
	Hemolysin III	--
	The repeat in toxin (RTX);	rtxA; rtxB; rtxC; rtxD; rtxE; rtxH
	Thermostable hemolysin (TH)	--
	Exotoxin A (ETA)(Pseudomonas)	toxA
**Antiphagocytosis**	Capsular polysaccharide(Vibrio)	rmlC
**Immune evasion**	Capsule(Acinetobacter)	--
**Others**	O-antigen(Yersinia)	--

Table S3: Plasmid-associated genes identified in strain A-9^T

Strain	Accession Number	Product	Protein ID
A-9^T	XNM42563.1	Plasmid recombination protein, partial	2894341482
	XNM42562.1	Hypothetical protein ACLMAI_24355	2894341481
	XNM42561.1	Type IV toxin-antitoxin system AbiEi	2894341480
	XNM42560.1	Nucleotidyl transferase AbiEii/AbiGii toxin family	2894341479
	XNM42559.1	PP2C family protein-serine/threonine phosphatase	2894341478
	XNM42558.1	ParA family partition ATPase	2894341477
	XNM42557.1	Plasmid partition protein ParG	2894341476
	XNM42556.1	Hypothetical protein ACLMAI_24325	2894341475
	XNM42555.1	Plasmid replication protein RepB	2894341474
	XNM42570.1	Replication initiation protein	2894341490
	XNM42569.1	Hypothetical protein ACLMAI_24390	2894341489
	XNM42568.1	Type II toxin-antitoxin system RelE/ParE family toxin	2894341488
	XNM42567.1	CopG family ribbon-helix-helix protein	2894341487
	XNM42566.1	Type IV secretion system protein	2894341486
	XNM42565.1	Plasmid replication protein RepB	2894341485
	XNM42564.1	Hypothetical protein ACLMAI_24365	2894341484
	XNM42577.1	MbeD/MobD family mobilization/exclusion protein	2894341498
	XNM42576.1	Type II toxin-antitoxin system CcdA family antitoxin	2894341497
	XNM42575.1	CcdB family protein	2894341496
	XNM42574.1	Hypothetical protein ACLMAI_24415	2894341495
	XNM42573.1	Replication initiation protein	2894341494
	XNM42572.1	Hypothetical protein ACLMAI_24405	2894341493
	XNM42571.1	MobC family plasmid mobilization relaxosome protein, partial	2894341492
	XNM42578.1	MbeB family mobilization protein	2894341499
